# A case of TM infection with challenging differential diagnosis from lymphoma post-renal transplant

**DOI:** 10.1186/s12879-023-08912-7

**Published:** 2023-12-19

**Authors:** Sulin Luo, Xingxia Wang, Xue Ren, Yamei Cheng, Luying Guo, Pengpeng Yan, Junhao Lv, Xinhui Su, Jia Shen, Kui Zhao, Ke Sun, Jianghua Chen, Rending Wang

**Affiliations:** 1 Kidney Disease Center, the First Affiliated Hospital, School of Medicine, Zhejiang University, Hangzhou, China; 2 Key Laboratory of Kidney Disease Prevention and Control Technology, Hangzhou, Zhejiang Province China; 3 National Key Clinical Department of Kidney Diseases, Hangzhou, China; 4 Institute of Nephrology, Zhejiang University, Hangzhou, China; 5 Zhejiang Clinical Research Center of Kidney and Urinary System Disease, Hangzhou, China; 6 Department of Nephrology, 903rd Hospital of PLA, Hangzhou, China; 7 Department of Nephrology, Huzhou Central Hospital, Huzhou, Zhejiang Province China; 8 Department of Nuclear Medicine, PET Centre, First Affiliated Hospital, School of Medicine, Zhejiang University, Hangzhou, China; 9 Department of Pathology, First Affiliated Hospital, School of Medicine, Zhejiang University, Hangzhou, China

**Keywords:** Talaromycosis marneffei, *Talaromyces marneffei* infection, Kidney transplant, Lymphoma, Gastrointestinal bleeding

## Abstract

**Background:**

Lymphomas involving the gastrointestinal tract may be manifested as anti-inflammatory tract bleeding, abdominal lymph node enlargement, or even perforation of the gastrointestinal tract. After organ transplantation, the likelihood of post-transplant lymphoproliferative disorders increases, and some rare infections may also appear.

**Case presentation:**

Herein, we report a living transplant patient with talaromycosis marneffei (TSM) or *Talaromyces marneffei* (TM) infection with gastrointestinal hemorrhage and systemic lymph node enlargement, which presented clinically as lymphoma.

**Conclusion:**

This case is TSM in a kidney transplant patient, confirmed by lymph node biopsy and blood culture. The patient discharged from hospital successfully under the treatment of antifungal therapy and immunosuppressive therapy. Physicians should be aware that TSM can mimic lymphoma, and early diagnosis and treatment can benefit the outcomes.

## Introduction

The likelihood of post-transplant lymphoproliferative disorders (PTLDs), such as lymphoma, increases significantly after organ transplantation due to the use of immunosuppression. Gastrointestinal lymphoma is characterized by gastrointestinal bleeding, perforation, and enlargement of lymph nodes [1]. Transplantation increases the likelihood of infection with opportunistic pathogens which can sometimes present similarly to lymphoma [2–4]. Herein, we report a case of talaromycosis marneffei (TSM) or *Talaromyces marneffei* (TM) infection in a kidney transplant patient mimicking lymphoma to provide evidence for physicians.

TM is a rare pathogen with a biphasic-type temperature. Among its two phases, namely, the hyphal phase at 25 °C and the yeast-like phase at 37 °C, only the latter is pathogenic [5]. Although it has been nearly half a century since TSM was first reported in 1973, it has not attracted the attention of researchers [6]. TSM is relatively more prevalent in tropical regions, such as southern China, India, Thailand, Vietnam, and Southeast Asia [7]. Immunocompromised patients, especially AIDS patients, including patients who are immunosuppressed after organ transplantation, are more prone to TSM [8]. Common clinical manifestations of TSM are fever, chills, cough, expectoration, weight loss and fatigue, superficial lymphadenopathy, hepatosplenomegaly, subcutaneous nodules, and bone and joint damage, among others [5]. It leads to a significant increase in white blood cell count and varying degrees of anemia. On the other hand, the clinical manifestations of TSM can mimic those of other infections or malignancies. Therefore, it is difficult for clinicians to identify. Herein, the case discussed can provide diagnosis and treatment value for clinicians.

## Case description

A 33-year-old man with a 10-year history of ABO-compatible kidney transplantation due to IgA nephropathy presented with left lower abdomen and back pain for more than 10 days. The patient was on an immunosuppressive therapy regimen that included tacrolimus, mycophenolate mofetil, and prednisolone and maintained with serum creatinine at 110–120 μmol/L. Until more than 10 days ago, the patient had pain in the left lower abdomen and back without any other signs, including chills, fever, chest tightness, shortness of breath, nausea and vomiting, tenesmus, diarrhea, and melena. The vital signs revealed a body temperature of 37.0 °C, blood pressure of 126/92 mmHg, pulse of 103 beats per minute, and respiratory rate of 18 breaths per minute. Physical examination revealed bilateral cervical lymphadenomegaly.

**Fig. 1:**
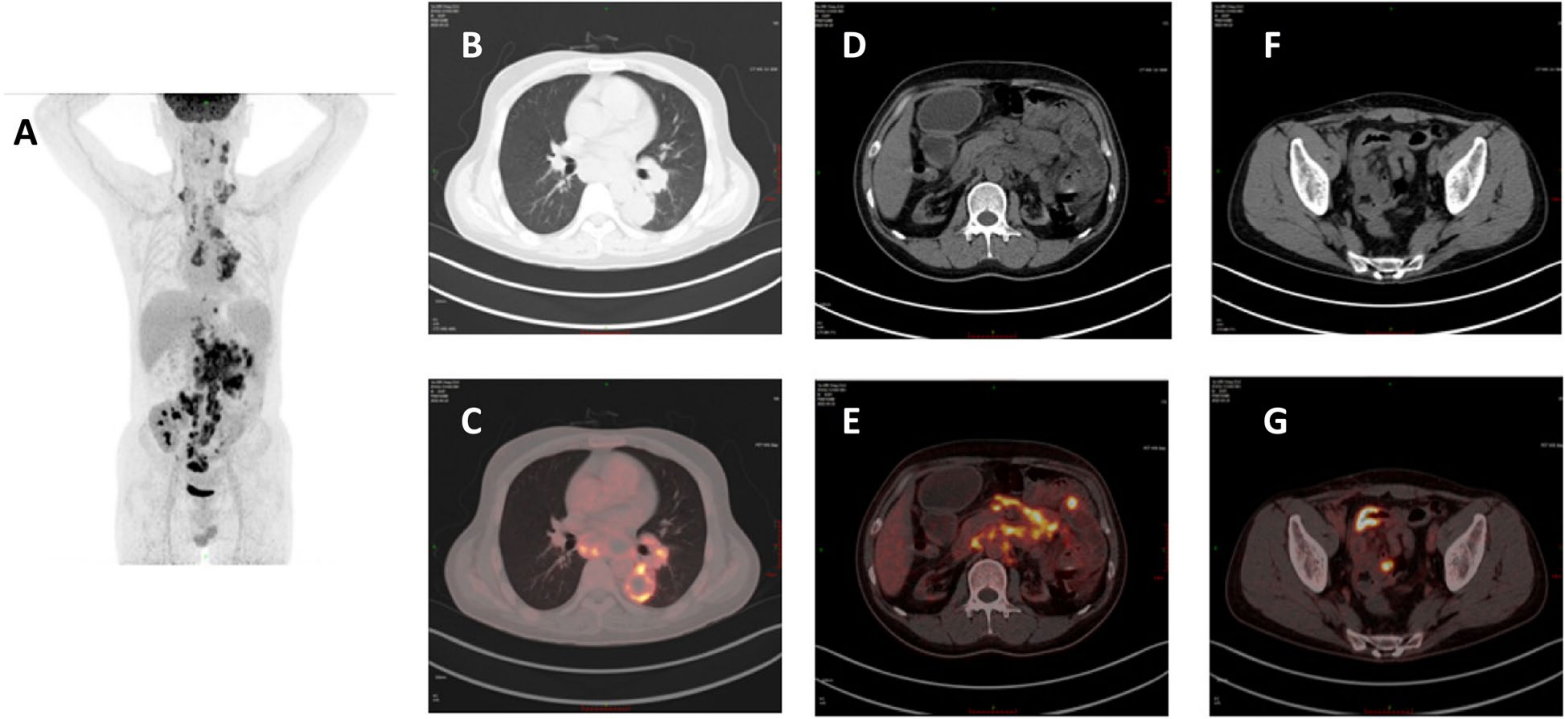
Head-Body PET Examination + Glucose Measurement (18F-FDG) (**A**, **C**, **E**, **G**) showed multiple lymph nodes were enlarged, the left hilar and mediastinal lymph nodes were enlarged, soft tissue density clumps of the dorsal and posterior basal segments of the left lower lobe, (**B**, **C**), multiple lymph nodes were enlarged in the retroperitoneal and superior mesangial and mesangial roots (**D**, **E**), multiple parts of the small intestine in the abdominal-pelvic cavity are thickened and FDG metabolism increased abnormally (**F**, **G**)

Laboratory results at the hospital reflected increased white blood cell percentage, hypohemoglobin, decreased percentage of lymphocytes, elevated levels of C-reactive protein, and increased creatinine. The cytomegalovirus antibody and deoxyribonucleic acid (DNA) was negative, and the Epstein-Barr virus (EBV) antibody and DNA were also negative. Various tumor markers were negative, while the fecal occult blood test was positive. In addition, alterations were observed in hepatic function measures: total protein and albumin were decreased (Table 1). A plain CT scan of the whole abdomen revealed multiple enlarged lymph nodes in the retroperitoneum and at the root of the mesentery. Furthermore, a plain CT scan of the lungs showed the left lower lung occupied space, enlarged left hilar and mediastinal lymph nodes, proliferative lesions in both lungs, and thickening of the left pleura (Fig. 1). B-ultrasound revealed multiple enlarged lymph nodes in the bilateral neck, and retroperitoneum was detected with splenomegaly, atrophy in two naive kidneys, and the transplanted kidney was normal with perfect blood perfusion, while blood flow of the mesenteric artery had a smooth flow.

**Table 1:** Clinical parameters at different time points

Clinical laboratory results after admission						
Measure	Reference range	On admission	1 week	1 month	4 months	8 months
White-cell count (10E9/L)	4.0–10.0	6.04	4.05	3.02	7.46	5.59
Absolute neutrophil count (10E9/L)	2.0–7.0	4.92	3.23	2.17	4.77	3.01
Neutrophil(%)	50.0-70.0	81.4↑	79.8↑	67.7	63.9	53.9
Absolute lymphocyte count (10E9/L)	0.8–4.0	0.52↓	0.35↓	0.7↓	1.82	1.89
Lymphocyte(%)	20.0-40.0	8.6↓	8.6↓	21.9	24.4	33.8
Red-cell count (10E12/L)	4.09–5.74	3.25↓	2.83↓	2.41↓	4.50	4.11
Hemoglobin (g/L)	131–172	85↓	74↓	64↓	123↓	114↓
Platelet count (10E9/L)	83–303	265	227	237	260	210
C-reactive protein (mg/L)	0.00–8.00	63.03↑	54.1↑	ND	ND	ND
Βeta-1,3-D glucan (pg/ml)	1-60	ND	ND	< 10	ND	ND
Sodium (mmol/L)	137-147	137	138	136↓	141	141
Potassium (mmol/L)	3.50–5.30	3.72	4.24	4.22	4.13	4.23
Chloride (mmol/L)	99-110	101	104	107	108	108
Blood urea nitrogen (mmol/L)	3.10-8.00	ND	9.72↑	3.41	10.96↑	13.08↑
Creatinine (μmol/L)	57-97	224↑	245↑	163↑	204↑	226↑
Total protein (g/L)	65.0–85.0	58.8↓	50.8↓	54.8↓	63.2↓	64.6↓
Albumin (g/L)	40.0–55.0	35.9↓	31.9↓	36.1↓	42.5	43.7
Alanine aminotransferase (U/L)	9-50	12	11	9	8↓	9
Aspertate Aminotransferase (U/L)	15-40	15	14↓	11↓	14↓	12↓
Total bilirubin (μmol/L)	0.0–26.0	6	3.5	7.9	4.1	6.6
Thrombin time(s)	14.5–21.5	ND	16.8	16.1	ND	ND
Prothrombin time(s)	10.0–13.5	ND	15.7↑	13.3	ND	ND
D-dimer (μg/L FEU)	0–700	ND	840.00↑	6550.00↑	ND	ND

The patient received cefoperazone and sulbactam anti-inflammatory treatment, with which the pain was slightly alleviated. However, after 1 week of treatment, the patient still had positive fecal occult blood, anemia, and persistent abdominal pain. Other laboratory results showed the same results of on admission (Table 1). The patient had gastrointestinal bleeding (black stool), abdominal pain, and other discomforts, and the possibility of tumor invasion was considered. He was given tramadol for the pain, somatostatin to inhibit gastrointestinal bleeding, meropenem for anti-infection, enteral nutrition, and other symptomatic and supportive treatment.

Afterward, a positron emission tomography–computed tomography (PET/CT) scan revealed that the abdominal cavity and thoracic superficial multiple lymphatic enlargement, pulmonary and intestinal involvement, malignant tumor metastasis was highly considered, and lymphoma should be considered first (Fig. 1), and lymph node biopsy was recommended to confirm the diagnosis.

**Fig. 2:**
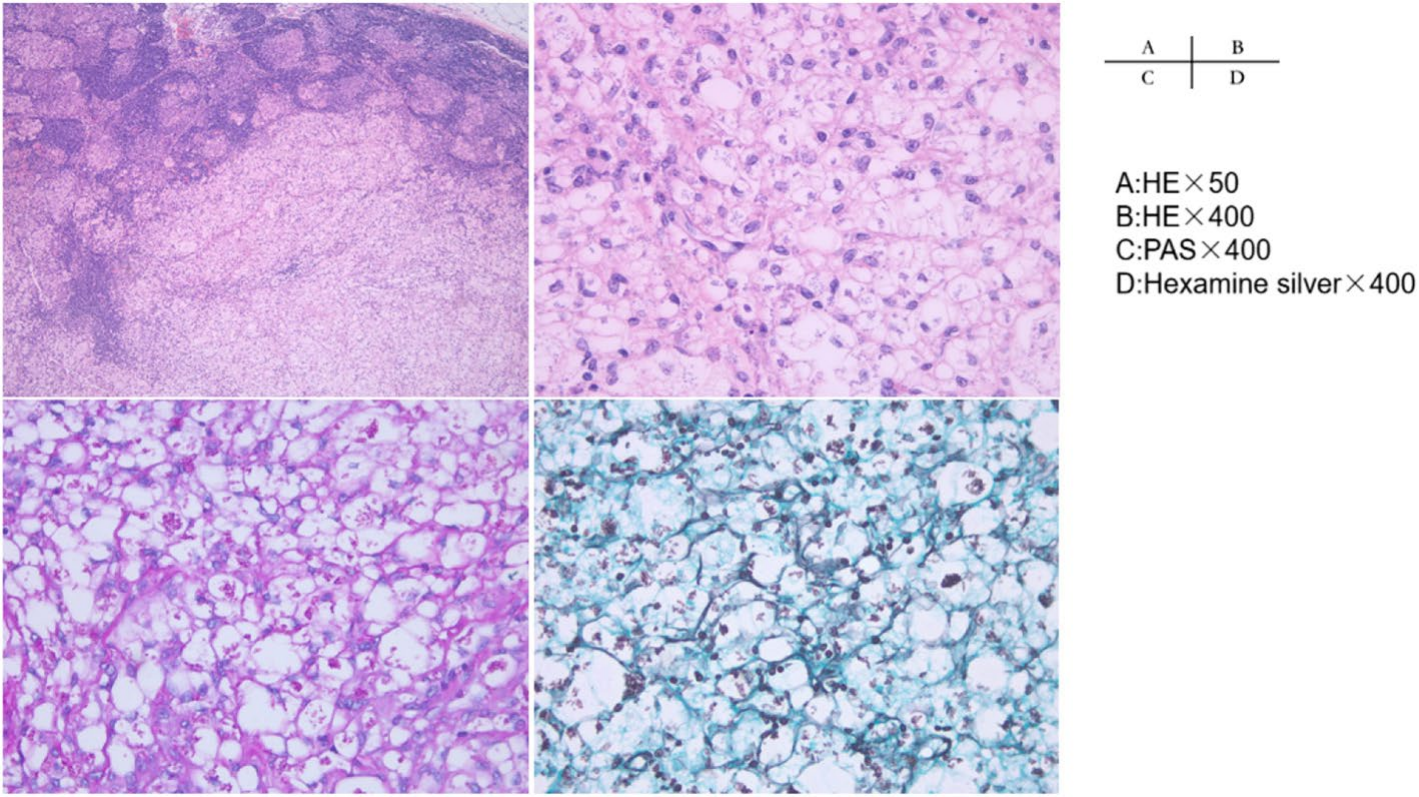
H&E staining showed that (left neck) massive hyperplasia of histiocytes / foam cells in lymph nodes with non-caseous necrosis, and particles in cytoplasm (**A**) (× 50 magnification) and higher magnification (**B**) (× 400 magnification), consistent with special infectious lesions (possibly TM or histoplasmosis). PAS staining revealed some intracellular and extracellular yeasts distributed in the lymph node (**C**) (× 400 magnification). Hexamine silver showed yeasts with positive (**D**) (× 400 magnification)

The patient’s pain was not well controlled by tramadol. PET-CT suggested lymphatic metastasis in posterior peritoneum, and the possibility of intrusion into the abdominal nerve plexus was considered. Oxycodone sustained-release tablet 10 mg q12h and pregabalin capsule 75 mg bid were recommended for pain relief. Subsequently, colonoscopy was subsequently recommended, but the patient’s temperature was elevated and hemoglobin was stable, and the patient refused the colonoscopy and was not performed. Pathological lymphadenectomy showed: (left neck) lymph nodes saw massive proliferation of histiocytes/foam cells, accompanied by non-caseating necrosis, and granular substances in the cytoplasm, and special examination: TB-PCR (−), CD20 (B cell +), CD3 (T cell +), CD21 (FDC +), CD23 (FDC +), EBER (−), CD68 (+ weak), Ki-67 (+ 20%), acid resistance (−), silver hexanamine (+), PAS (+), TB (FISH) (−), fungal (FISH) (+), diagnosing special infectious lesions (TM; Fig. 2). Blood cultures suggested TSM. The diagnosis was finally confirmed as TM infection invading the small intestine leading to gastrointestinal bleeding.

Itraconazole capsules 200 mg bid symptomatic treatment were given, in addition to somatostatin, tranexamic acid injection, fensulfame injection, and vitamin K1 while reducing the dosage (1 mg twice a day to 0.5 mg twice a day) and the concentration (from 6.6 ng/ml to 5.0 ng/ml) of tacrolimus and cessation of mycophenolate mofetil. One month after admission routine examination results (Table 1) demonstrated that the patient was essentially back to normal and had some anemia. Furthermore, his serum creatinine was 163 μmol/L. The follow-up procedure involved regular antifungal therapy and immunosuppressant therapy. After 8 months of antifungal treatment, the lungs basically resolved.

## Discussion

We report herein a rare case of disseminated TSM after kidney transplantation in a patient who presented with abdominal pain, multiple superficial and intraperitoneal lymphadenopathy, and gastrointestinal bleeding, highly suspected of lymphoma. The patient was confirmed with TSM with cervical lymph node biopsy after PET/CT suspected of lymphoma and blood culture. Immediate and effective treatment with itraconazole achieved desired result.

The incidence of PTLD has increased significantly and its clinical manifestations mimic TSM, and pathological biopsy may be the main distinguishing method. Disseminated TSM has a high fatality rate, and the mortality rate can reach 24–33% after antimicrobial treatment [8]. However, due to the hidden onset, patients and doctors might not pay much attention to it in the early stage, so it is easy to misdiagnose and delay treatment [9]. TM can spread to multiple systems through the blood, such as the respiratory system, digestive system, reticuloendothelial system, and easily invade the mononuclear macrophage system [8]. Due to the use of immunosuppressants after organ transplantation, the incidence of PTLD has increased significantly, reaching 5.7% [10]. This patient presented with abdominal pain, superficial and abdominal multiple lymphadenopathy, and gastrointestinal bleeding. PTLDs, especially lymphoma, are considered highly dangerous. Lymphoma is a malignant tumor originating from the lymphatic hematopoietic system. Its primary manifestations are painless lymphadenopathy, hepatosplenomegaly, and all tissues and organs of the body can be involved, which has similar symptoms to TSM but accompanied by systemic symptoms such as fever, night sweats, weight loss, and itching. The main distinguishing point is the pathological findings. TSM’s pathological features are some intracellular and extracellular yeasts. However, the basic pathomorphological change of Hodgkin’s lymphoma is to see diagnostic Reed–Sternberg cells and their variant cells in the background of mixed proliferation of various inflammatory cells [11, 12]. Hence, this case was confirmed as a TSM by biopsy.

As far as is known, lymphoma is difficult to distinguish from EBV and tuberculosis. Lymphoma is closely associated with EBV. EBV, originally discovered through its association with Burkitt lymphoma, can cause lymph node enlargement, fever, sore throat and other clinical manifestations, R-S cells can be seen under the microscope and is now etiologically associated with a wide range of lymphoproliferative lesions and B, T, and NK cell-derived malignant lymphomas [13]. Lymphoma is often misdiagnosed as tuberculosis, prolonging treatment and potentially adversely affecting patient outcomes as the disease progresses. Existing tuberculosis guidelines for smear negative cases are unclear about when to consider an alternative diagnosis [14]. Lymph node biopsy, bone marrow biopsy and immunochemistry are very effective in differentiating lymphoma from tuberculosis and EBV [15]. It is rare for lymphoma and TSM to be indistinguishable from each other.

The main causes of gastrointestinal bleeding are digestive ulcer, stress ulcer, drugs, esophagus and gastric varices rupture bleeding, gastric cancer and so on [16, 17]. The causes of gastrointestinal bleeding in kidney transplant patients may be previous history of digestive tract (peptic ulcer), stress ulcer caused by surgery, and gastrointestinal mucosa damage caused by taking a large amount of steroids and immunosuppressants after kidney transplantation [18, 19]. Gastrointestinal bleeding due to infection in kidney transplant patients is uncommon, and bleeding due to TSM is even rarer.

Considering the clinical manifestation features (stomachache, intestinal bleeding, and extensive lymphadenopathy), the differential diagnosis of gastrointestinal tumors, tuberculosis, lymphoma, and Crohn’s disease should be considered. Symptoms of TSM in 18.8 to 31% present with gastrointestinal bleeding, primarily affecting the colon [9]. The best differential diagnosis is colon endoscopy and biopsy [9]. Intestinal bleeding may first be caused by TM affecting the intestinal tract (multiple ulcers). As PET/CT revealed TSM mainly involved the small intestine, we confirmed TSM after PET/CT and biopsy, but unfortunately the patient has not received endoscopy and biopsy, which is also the limitation.

For confirmed infection, the most common reason for high mortality [20] is a late diagnosis and consequently no effective timely treatment. Furthermore, because it is bidirectional bacteria, culture temperature back affects the results. Blood next-generation-sequencing (NGS) may increase the early detection rate [21], and our unpublished data suggest that NGS can facilitate early detection in organ transplantation. Timely treatment can greatly improve patient outcomes [8]. Amphotericin B is the gold standard for systemic antifungal therapy [22]. However, the side effects and adverse reactions of this drug usually include stomach discomfort and decreased renal function. Combining with our experience, it has not been used for this patient. Multiple reports have confirmed that itraconazole exhibits good antifungal efficacy [23–25]. The patient received itraconazole with a good prognosis. Appropriate and timely diagnosis and early aggressive antifungal therapy can improve the clinical outcomes of patients. However, during the follow-up of the patient, creatinine was still maintained at about 200 μmol/L. We considered the increase in creatinine for the following reasons: First, due to the severity of the infection, we stopped mycophenolate and reduced the concentration of tacrolimus, which can lead to chronic rejection; Secondly, the patient subsequently resumed the dose of tacrolimus and the use of azole drugs, which have some renal toxicity in high concentrations. Unfortunately, there was no kidney biopsy for identification.

In summary, this rare case of TSM after transplantation can present with lymphoma. Therefore, early detection and early treatment can significantly improve the prognosis of patients, improve the quality of life, and increase the survival rate.

## Data Availability

The datasets used and/or analyzed during the current study available from the corresponding author on reasonable request.

## Abbreviations

TM: *Talaromyces marneffei*
TSM: *Talaromycosis* marneffei
AIDS: Acquired immunodeficiency syndrome
PTLD: Post-transplant lymphoproliferative disorders
DNA: Deoxyribonucleic acid
EBV: Epstein-Barr virus
